# A Step‐by‐Step Protocol From METASPACE to Biological Interpretation

**DOI:** 10.1002/jms.70072

**Published:** 2026-06-11

**Authors:** Abigail Moreno‐Pedraza, Brittney Gorman, Marija Velickovic, Max Bentelspacher, Jaime Barros, Dusan Velickovic, Christopher R. Anderton

**Affiliations:** ^1^ Earth and Biological Sciences Directorate Pacific Northwest National Laboratory Richland Washington USA; ^2^ Division of Plant Science and Technology University of Missouri Columbia Missouri USA

## Abstract

Mass spectrometry imaging (MSI) represents an exceptional tool for exploring complex biological systems spatially at the molecular level. However, its multidimensional nature and large data outputs make it challenging to extract meaningful biological insights. Advancements such as the METASPACE platform allow researchers to efficiently process, annotate, and interpret MSI datasets by leveraging machine learning and a cloud‐based infrastructure. In this tutorial, we present a detailed and user‐friendly R‐pipeline designed to help METASPACE users navigate untargeted metabolomic annotations and translate them into practical biological insights, particularly in complex systems. This approach has broad potential applications, including diagnostics, drug discovery, environmental, and ecological research. We envision this pipeline will be particularly useful for newcomers to MSI and encourage experienced users to customize and extend it to meet more advanced analytical needs.

## 1 Introduction

Mass spectrometry imaging (MSI) has become a very popular analytical technique for simultaneously visualizing the distribution and the relative abundance of molecules in biological tissues and microbial systems [1, 2]. Among the diverse sets of molecules commonly analyzed, metabolites and lipids are among the most popular. Their study is crucial to understanding ecological interactions and biochemical processes within living organisms [3]. Research on small molecules has significant practical applications, such as natural product discovery [4], disease diagnostics [5], biofuel production, and more. Visualizing their accurate localization and characterization is critical, particularly in natural product chemistry, biomarker research, and understanding their biological activity [6]. MSI plays a crucial role in advancing these areas by providing detailed insights into the molecular distribution. However, translating MSI measurements into biological conclusions remains challenging, largely because of accurate molecular identification and interpretation by the chemical diversity of complex samples and the large number of potential molecular candidates [7].

METASPACE is a cloud‐based MSI annotation engine and knowledgebase, which is a community‐populated repository that allows researchers to investigate and putatively annotate what has been imaged by different MSI instruments [8]. At the end of 2025, METASPACE contained more than 16 000 MSI datasets submitted from all over the world. Its popularity relies on its ability to annotate and visualize metabolites [9], lipids, and *N*‐glycans [10] from submitted MSI datasets; the only requirement is to upload data from a high‐resolution mass spectrometer in centroided mode and in the open format imzML [11]. Once the data are submitted, an MS1‐based metabolite annotation engine processes the data and provides molecular annotations on a pseudolevel 2 (putatively annotated compounds), based on the Metabolomics Standard Initiative [12], where detected molecular formulas are annotated from selected database(s)—including customs in‐house generated databases.

**FIGURE 1:**
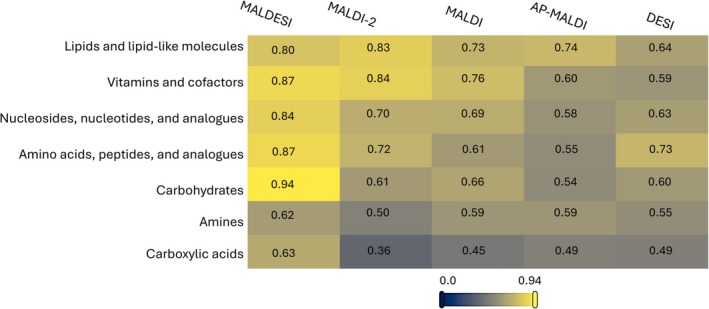
Heatmap based on METASPACE annotations illustrating detectability across chemical classes and ionization/desorption sources (Cividis colormap Ref [13]). Rows correspond to chemical classes, and columns represent ionization/desorption sources: matrix‐assisted laser desorption electrospray ionization (MALDESI), matrix‐assisted laser desorption/ionization (MALDI), laser post‐ionization MALDI (MALDI‐2), atmospheric‐Pressure (AP)‐MALDI, and desorption electrospray ionization (DESI). The color scale indicates the fraction of ions detected for each chemical class‐ionization/desorption source, with cell values reporting the fraction detected (range: 0–0.94), where 0.94 corresponds to 94% of ions detected (the maximum observed across chemical classes).

To date, METASPACE includes datasets from a wide variety of instruments and samples, enabling the detection and putative identification of an extensive range of metabolites and lipid molecules. Figure 1 illustrates the fraction of chemical classes detected from datasets across different ionization sources. Although METASPACE facilitates the annotation, users still face a major bottleneck after annotation: efficiently summarizing, comparing, and prioritizing results across datasets or experimental conditions.

Identifying how metabolite distributions change under different conditions is one of the primary objectives in most MSI experiments, and for an untargeted metabolomics approach, these analyses require assessing the distribution of hundreds or even thousands of ions [14]. Therefore, manual inspection of ion images or spreadsheet‐based handling of annotation tables is time‐consuming, difficult to standardize, and prone to inconsistencies, particularly when multiple datasets, sample types, or acquisition methods are involved.

**FIGURE 2:**
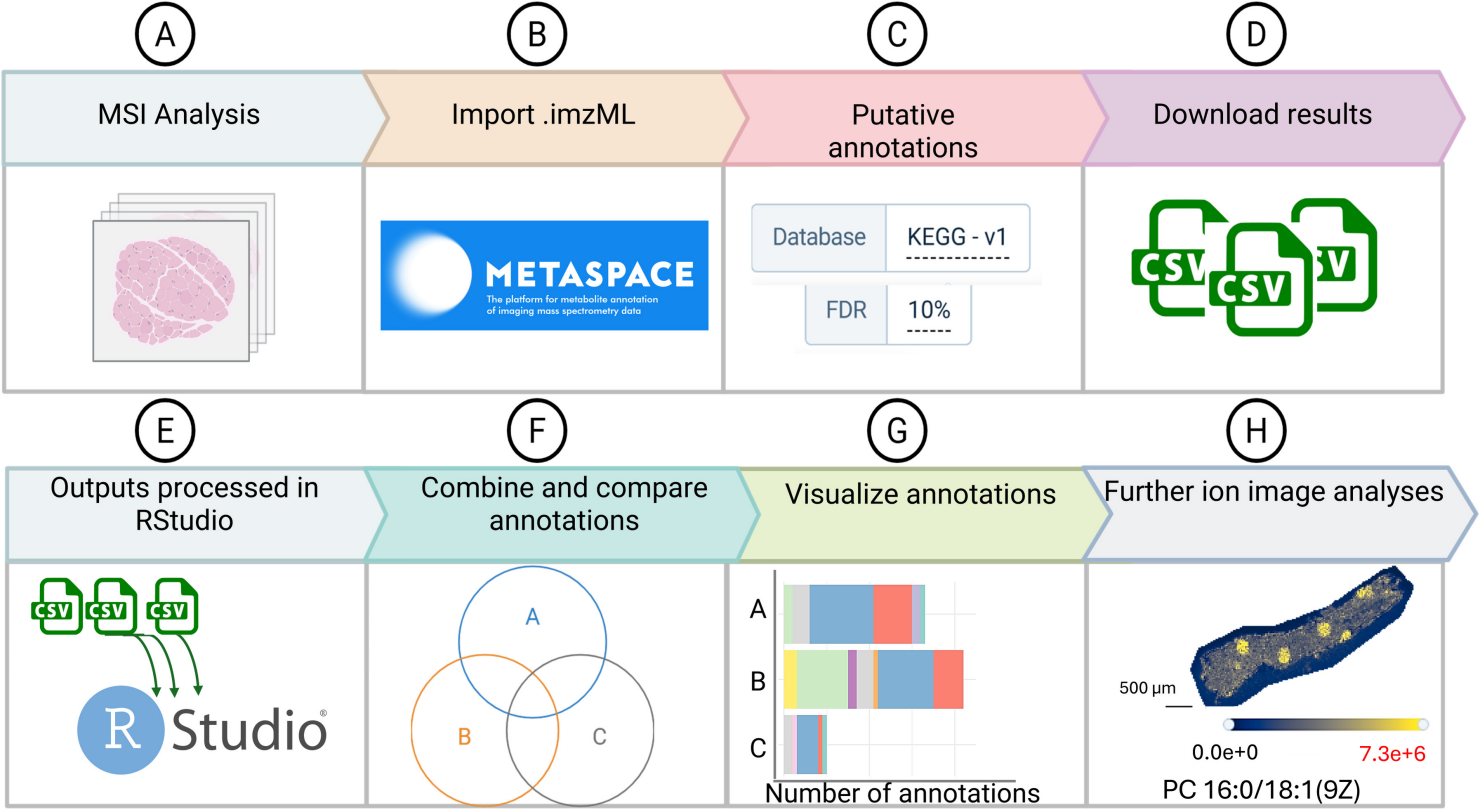
End‐to‐end workflow for MSI annotation to comparative analysis. (A–D) MSI data are acquired, converted/imported as imzML into METASPACE, evaluated across datasets, and annotation outputs are downloaded as CSV files. (E–H) CSV outputs are processed in RStudio to consolidate annotations across experiments, generate comparative summaries and visualizations, and produce a curated annotation set for targeted follow‐up, including ion‐image inspection in dedicated MSI software.

To address this gap, we present a practical, step‐by‐step tutorial and R‐based workflow that helps streamline the post‐METASPACE annotation of the MSI datasets. The goal of this protocol is to enable a rapid generation of interpretable results, condensed into summaries (e.g., shared vs. unique annotations and chemical class distributions), and produce refined annotation lists that can be directly used for targeted visualization and for further guidance into a deeper downstream analysis using dedicated MSI software (Figure 2). This approach is intended to lower the barrier for initial biological interpretation and to improve consistency and reproducibility when working with complex MSI datasets.

## 2 Materials and Methods

Samples from Users of the Environmental Molecular Sciences Laboratory (EMSL), from the United States National Institutes of Health Kidney Precision Medicine Project (KPMP), and published datasets available in METASPACE [15] were used as examples to showcase the use of the R‐script developed to facilitate an initial and easy inspection of datasets uploaded to METASPACE independently from the ionization source or mass analyzer used.

In Example S1, *Sorghum bicolor* stems were collected at two different developmental stages, 5 and 7 weeks, and metabolic profiles were analyzed in negative mode with matrix‐assisted laser desorption/ionization (MALDI) Fourier transform ion cyclotron resonance (FTICR) with a mass resolving power of 110 000 (at *m*/*z* 400), using *N*‐(1‐naphthyl) ethylenediamine dihydrochloride (NEDC) as the MALDI matrix, and metabolites were annotated against the KEGG [16], Core Metabolome [14], and NPA [17] databases. In Example S2, human kidney biopsies were obtained through the KPMP repository and cryosectioned at 7 μm for lipid analysis in positive mode using MALDI‐FTICR‐MSI (160 000 at *m*/*z* 400), dihydroxybenzoic acid (DHB) as the matrix, and were annotated against LIPID MAPS [18], SWISS Lipid [19], and KEGG databases in METASPACE. In Example S3, published datasets of Hunter et al. were downloaded from METASPACE to showcase the utility of the R‐script for rapid inspection of annotated molecules using a different ionization source. In this study, infrared (IR) matrix‐assisted laser desorption electrospray ionization (MALDESI) was used to analyze the mouse brain with a doped solvent and/or ice matrix and without. LIPID MAPS [18] was used for annotations. For data processing and visualization, we use the free software R [20] and the integrated development environment (IDE) RStudio [21].

## 3 Step‐by‐Step Protocol

**FIGURE 3:**
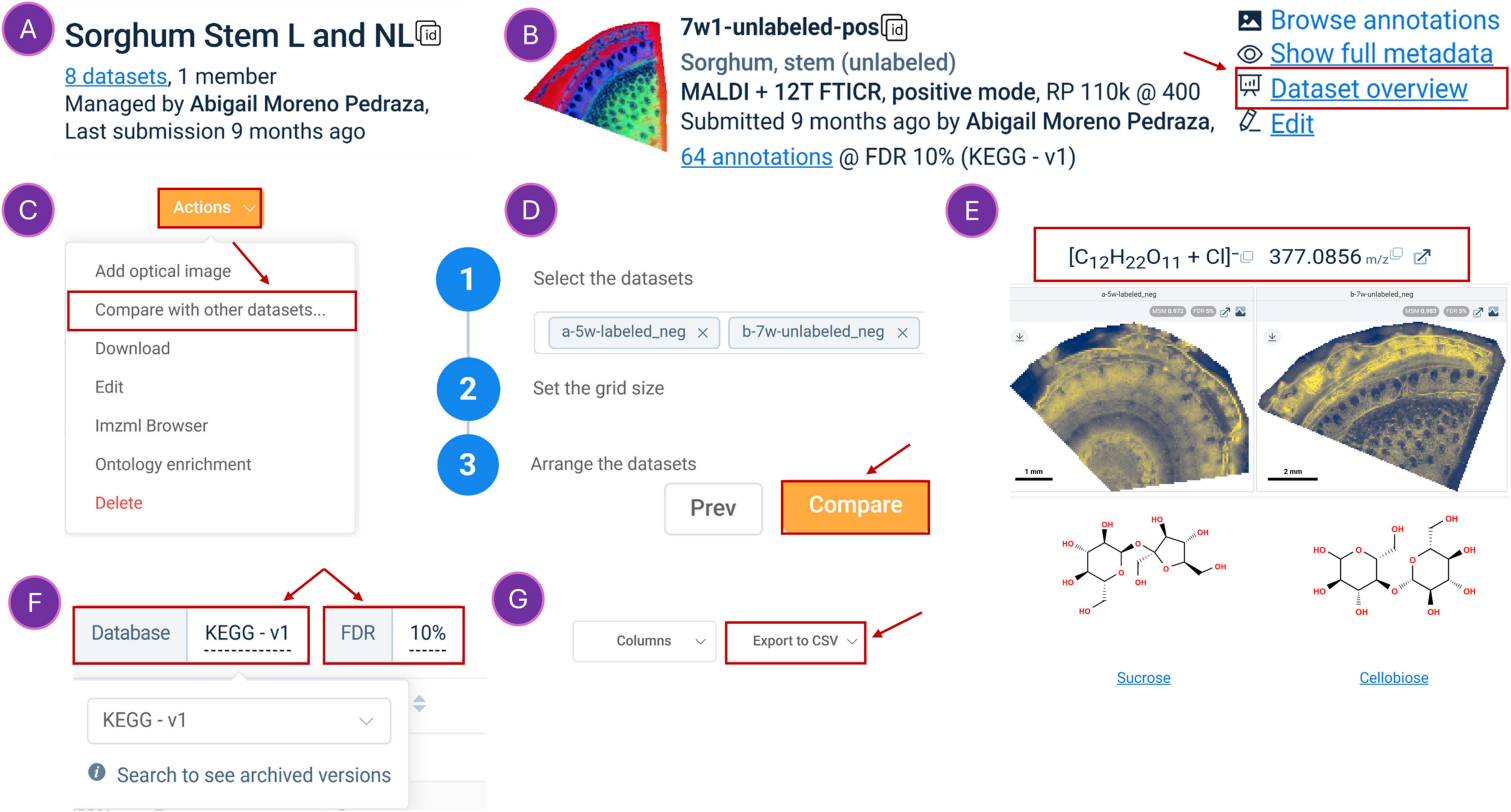
METASPACE step‐by‐step workflow for cross‐dataset comparison, using sorghum stem data as the comparison. (A) Select the project (here: Sorghum Stem L and NL). (B) Choose a dataset and open Dataset overview (red box). (C) From the Actions menu, select Compare with other dataset*s* (red box). (D) In the Datasets comparison view, select the datasets, arrange them for side‐by‐side display, and click Compare. (E) Example annotation showing a molecular formula assignment (red box) with the corresponding ion images across datasets; two candidate isomers are displayed. (F) Annotation settings can be adjusted from the table header, including the reference database (e.g., KEGG v1) and the FDR threshold (e.g., 10%). (G) Export the annotation table using Export to CSV (red box), repeat step *F* and *G* for exporting annotation using different databases.

An illustration of the METASPACE cross‐dataset comparison and the datasets export workflow is shown in Figure 3. Details are as follows:

1 Start in METASPACE. Open the project in METASPACE that contains the datasets to be analyzed and compared (Figure 3A).
2 Select datasets for comparison. Navigate to *dataset overview* and select a dataset. Use *dataset comparison* feature to select the samples to compare including replicates when available (Figure 3B–D). In the example shown, two sorghum stem datasets are compared.
3 Arrange and compare. Arrange the selected datasets in the comparison grid and click *Compare* (Figure 3D).
4 Review ion images and annotation table. A new window will display side‐by‐side ion images along with the annotation table. Each row in the table includes the ion images, the molecular formula, and the list of putative molecular features (Figure 3E).
5 Set database and threshold. In the annotation table header, set the database and define a False Discovery Rate (FDR). An FDR of 10% is recommended by Palmer et al. [22]. To calculate the FDR, a target‐decoy approach is used to estimate how many annotations are likely incorrect. This value is dependent on the database selected; therefore, it is best to select databases most relevant to the sample type. For example, the KEGG database at 10% FDR is used for Figure 3F.

6 Export annotation table. Click Export to CSV to download the annotation table. Repeat this step for any additional databases that should be included in the comparison (Figure 3G). Using multiple relevant databases can increase coverage; however, in targeted studies, a single specialized database may be sufficient. In Example S1, KEGG, CoreMetabolome, and Natural Product Atlas (NPA) databases were used, whereas in Example S3, only the LIPID MAPS database was selected because lipids were the primary molecular target for this study [15].

**FIGURE 4:**
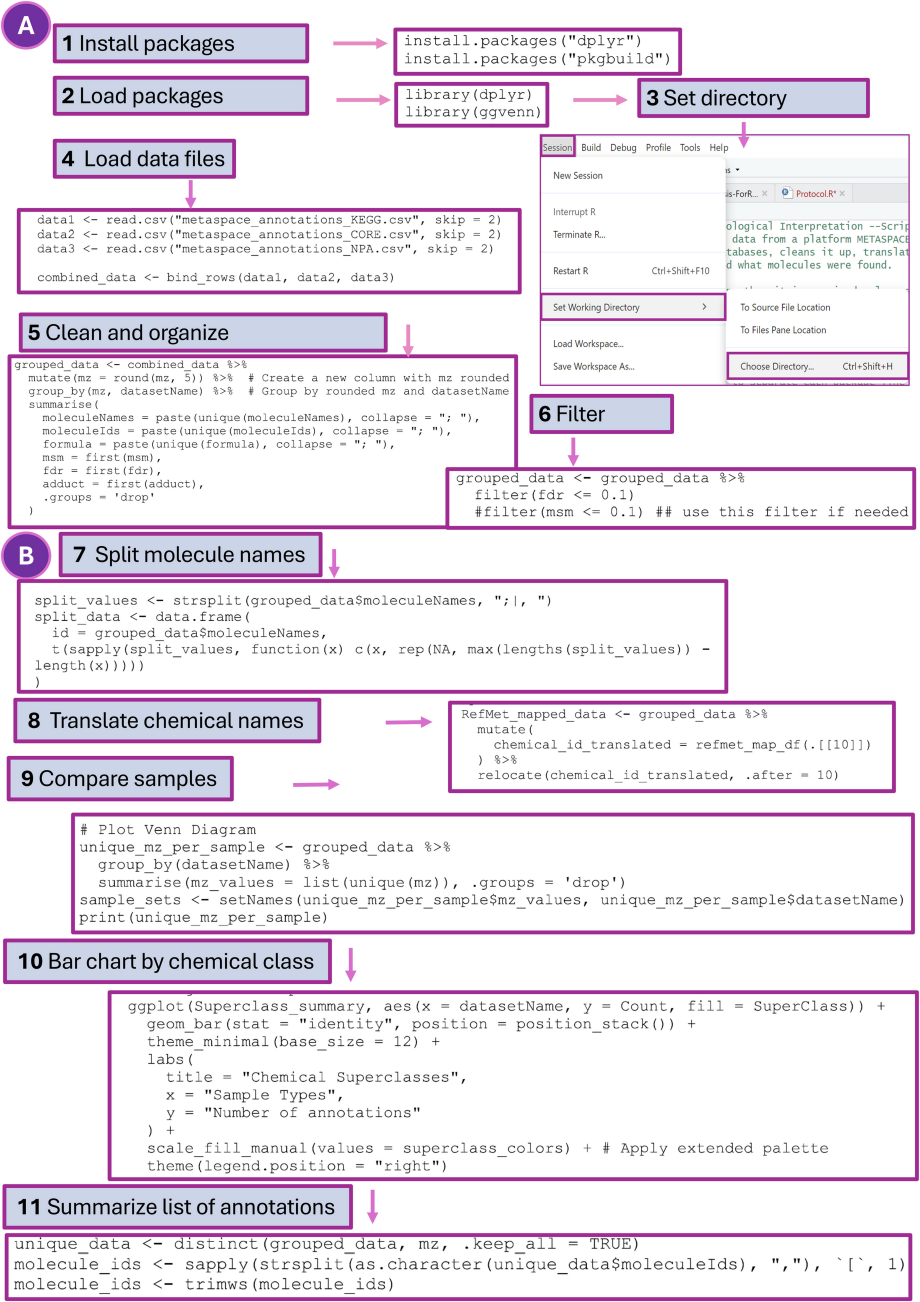
R‐script workflow for postprocessing METASPACE annotation. (A) Setup and data preparation: required R packages are installed and loaded, the working directory is set, METASPACE CSV files from multiple databases are imported and merged, and the combined table is cleaned/organized (m/z rounding, grouping by m/z × dataset, collapsing duplicate annotations) with optional filtering by FDR/MSM. (B) Downstream processing and reporting: multientry molecule‐name fields are parsed, chemical names are harmonized using RefMet, datasets are compared (e.g., overlap analyses), annotations are summarized by chemical class, and a refined, nonredundant annotation list is generated for subsequent targeted interpretation and visualization.

An illustration of the R/RStudio script usage: Import, clean, harmonize, classify, and compare are shown in Figure 4 and in supporting Video S1.

**FIGURE 5:**
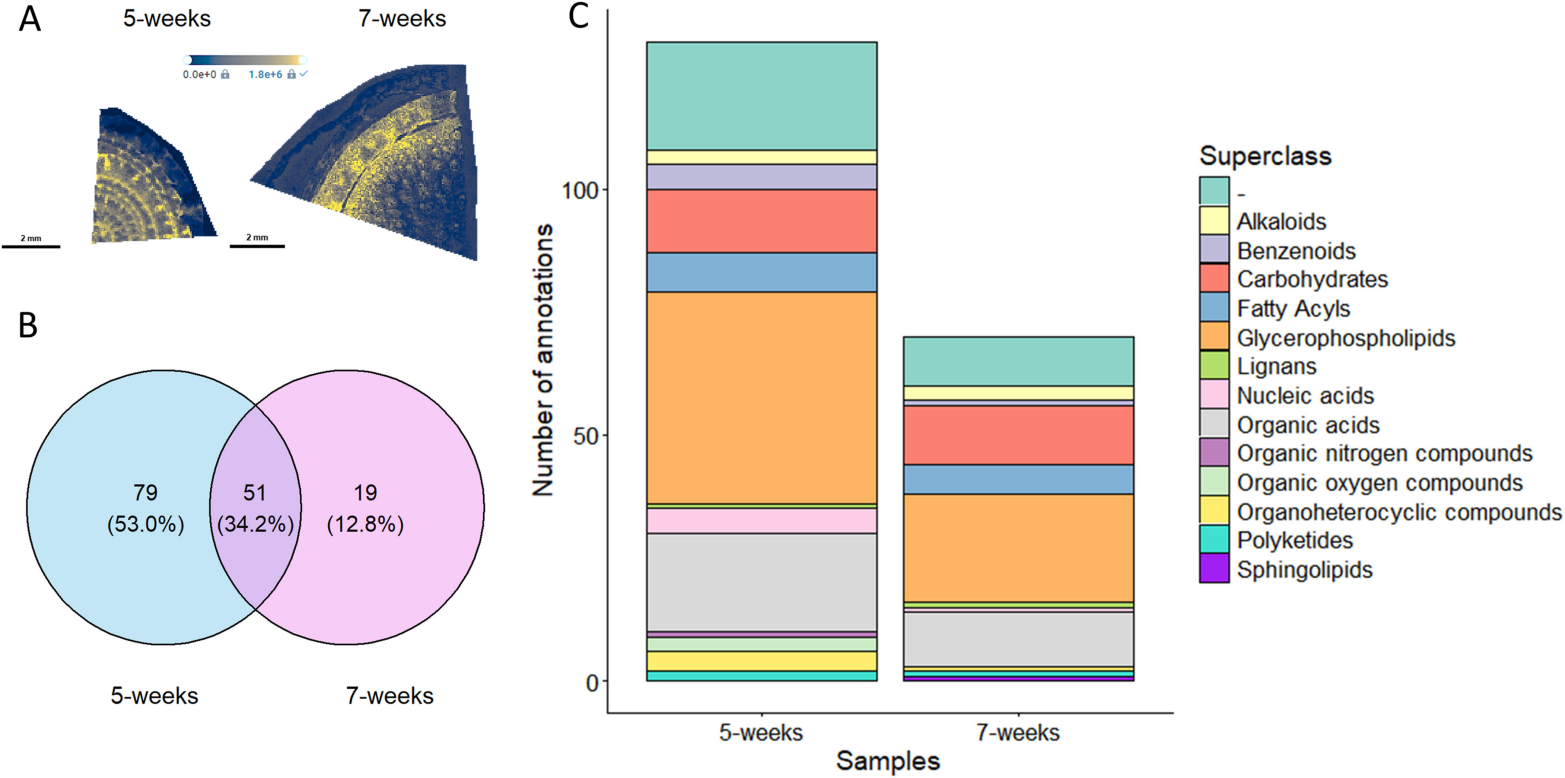
Comparison of MSI annotations between 5‐ and 7‐week sorghum stem samples. (A) Representative ion images for the same annotated feature shown in transverse stem sections at 5 and 7 weeks (scale bars indicate 2 mm; and intensity per the displayed color scale Cividis). (B) Venn diagram displays shared and sample‐specific annotated *m*/*z* features between the two datasets. (C) Superclass level classification of annotated features summarizing overall differences in the chemical composition between the samples.

**FIGURE 6:**
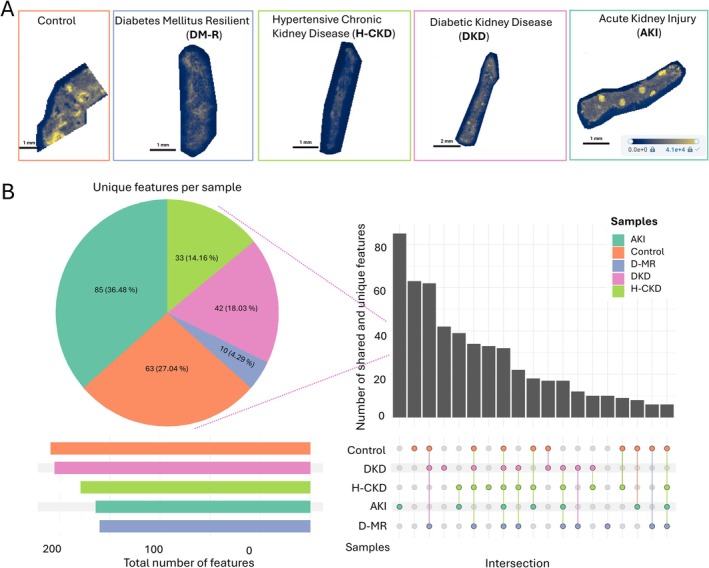
Cross‐sample comparison of MSI molecular annotations across kidney diseases. (A) MSI images of each sample type showing as an example the *m*/*z* 725.5568 corresponding to the molecular formula C_39_H_79_N_2_O_6_P and putatively annotated as C16 Sphingomyelin. Data are shown for control, diabetes mellitus–resilient (DM‐R), hypertensive chronic kidney disease (HCD‐K), diabetic kidney disease (DKD), and acute kidney injury (AKI) tissue. Total ion intensity normalization applied using the color scale Cividis. (B) Summary of ion features and their overlap across samples. The pie chart (left) reports the number (and percentage) of features that are unique to each sample type. The UpSet plot (right) summarizes the feature intersections: each vertical bar gives the number of features observed in the specific intersection indicated directly below by the connected filled dots (filled dot = feature present in that sample; gray dot = absent). Single‐dot columns therefore represent sample‐specific features, whereas multidot columns represent features shared by the connected set of samples only (e.g., pairs, triples, or higher order overlaps). Bars are ordered by intersection size (largest to smallest), allowing rapid identification of the shared feature sets across sample types.

**FIGURE 7:**
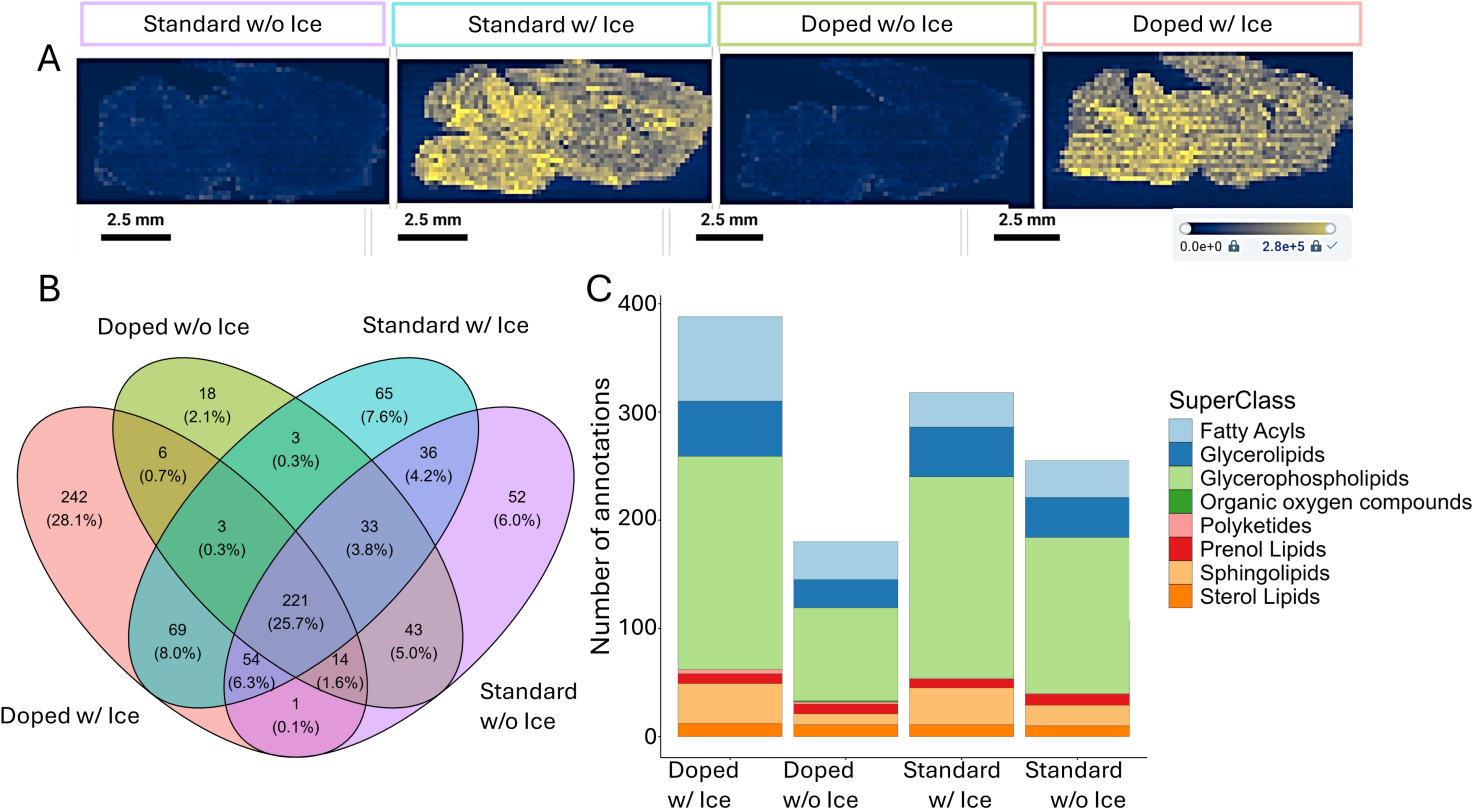
Comparison of METASPACE annotations across of MALDESI data using different sample/sampling‐preparation conditions. (A) Representative ion images for the same annotated feature with *m*/*z* 369.3516 across four conditions: standard spray condition without ice, and with ice, NH_4_F‐doped solvent without ice, and with ice (total ion count normalization). (B) Four‐way Venn diagram summarizing overlapping of unique *m*/*z* features among conditions; numbers and percentages indicate features unique to each condition and shared across intersections. (C) Stacked bar plot showing the number of annotated features per chemical superclass for condition.

7 Install and load packages. Open R Studio with the script provided. Install the required packages listed in Table 1; installation instructions are included in the script (Figure 4 A‐1). Once installed, load the libraries into the session (Figure 4 A‐2). Note that installation of R, Rstudio, RTools, and R‐packages is required only once.
8 Set working directory. Set your directory to the folder containing the downloaded CSV from METASPACE. To do this, go to the *Session* tab and select *Set Working Directory* and *Choose Directory* (Figure 4 A‐3).
9 Import the annotation files. Import the METASPACE annotation files into RStudio as shown in Figure 4 A‐4. Three CSV files can be combined using bind*_rows()*, creating a single consolidated annotation table stored in a new data frame named: *combined_data*. (or another name of your choice).
10 Clean and deduplicate. The combined frame contains duplicated annotations and multiple columns. This step cleans and organizes the data frame (Figure 4 A‐5). Rounding the *m*/*z* values to four decimal places, this can be adjusted depending on the mass analyzer used. Removing unnecessary columns and grouping rows that share both *m*/*z* and *datasetName* column. Collapsing redundant entries by merging multiple moleculeNames into a single entry separated by semicolon “;”. The clean results are saved in a new data frame in the example named *grouped_data*. Additional columns can serve as filters if needed. For example, an artificial intelligence approach embedded in METASPACE automatically distinguishes between “on‐sample” (biological) and “off‐sample” (background/matrix) images [23]. This approach is particularly suited for MALDI images. The use of the off‐sample column in MALDI leads to metabolite identification of only endogenous molecules within the samples. However, the off‐sample METASPACE approach is not well suited for all ionization sources, as the background signals for ambient ionization sources can be very different. Manually check the data to see how well this works for any ambient ionization data before proceeding, and use additional tools in the case the off‐sample METASPACE tool fails [24].
11 (Optional) apply a (Metabolite Single Match) MSM filter. The annotation table included a column called MSM score, calculated using METASPACE rule‐based algorithm that evaluates three distinct spatial and spectral criteria. MSM ranges from 0 to 1, with higher values indicating better agreement with theoretical isotope patterns [14]. The use of this filter is optional and can be used as follows: filter (MSM ≥ 0.4), which retains annotations that exceed or are equal to a MSM 0.4, which also fall into 5 or 10% FDR. To disable this filter, simply comment on the line using pound sign “#,” as shown in Figure 4 B‐6.
12 Prepare molecule names for classification. To enable chemical classification, the moleculeNames column must be split. This column contains candidate names from different databases separated by semicolons “;” and multiple candidates within a database separated by commas “,”. METASPACE provides putative identifications but cannot distinguish isomers (or isobars without sufficient mass resolution); a single *m*/*z* can have several candidate annotations (Figure 3E).
13 Harmonize molecule names. For classification, the script uses the first candidate name (Name1) (Figure 4 B‐7). Name harmonization is performed using the RefMet tool [25], which standardizes metabolite and lipid names using curated nomenclature developed by the NIH Metabolomics Workbench [26]. RefMet harmonizes metabolite and lipid names and classifies them based on their chemical taxonomies using both Classyfire [27] and LIPID MAPS [28] (Figure 4 B‐8). Using a standardized naming system improves consistency and interpretability. Before harmonization, review the split name lists to ensure that the first entry (Name1) is appropriate; users may optionally edit or remove unlikely annotations. Additional name columns (e.g., *Name2*, and *Name3*) can be included if desired. Confirm that the chemical name used for harmonization resides in the correct column position as shown in Figure 4 B‐8 and in Video S1, ensuring that RefMet classifications are appended accurately.
14 Comparing samples (Figure 4 B‐9). This section of the script facilitates the comparison between the unique and shared *m*/*z* features across the datasets. A graphic representation helps to quickly identify the number of *m*/*z* that are unique for a sample type or treatment and the total number of features that are shared among them, reducing the time spent manually reviewing images in METASPACE. Example S1 shows results from two sorghum stem samples using a Venn diagram. Example S2 compares five kidney biopsies using an UpSet plot. Example S3 presents a four‐sample comparison using a Venn diagram. Venn diagrams are particularly suitable for groups of up to four sample types; for more complex designs, UpSet plots [29] are recommended as shown in Example S2.
15 Export comparison results. After identifying unique and shared features, a new CSV file can be exported (for Venn diagram) or an Excel file (for Upset plots). For Venn diagram (Example S1 and Example S3), two new columns are added to the data frame: *source* and *status*. The *source* column contains the sample name (datasetName) in which the *m*/*z* appears. The *status* column shows whether the *m*/*z* is *unique* or *shared*. For Upset plots (Example S2), the script generates an .xlsx file containing three sheets: *Unique*, *Shared Among All*, and *Shared Between Groups*, each with a binary presence/absence matrix. Additionally, three summary columns: *SharedGroups*: sample containing the *m*/*z*, *NumGroups*: number of groups where the *m*/*z* appears, and *Category*: classification if the *m*/*z* is unique or shared. This structured output simplifies data organization and downstream analysis.
16 Visualize chemical composition. The script produces stacked bar plots summarizing the number of features per superclass, class, or subclass for each sample type (Figure 4 B‐10). These visualizations provide a quick overview of chemical distribution patterns and are particularly valuable in untargeted metabolomic MSI experiments. Each plot can be customized by selecting which taxonomic level (class, superclass, or subclass) to display.
17 Export a final nonredundant feature list. The final step in this script exports a consolidated list of nonredundant *m*/*z* features and their putative molecule annotations as a new CSV file (Figure 4 B‐11). This list is useful for downstream imaging analyses because it represents annotated *m*/*z* features present across all samples. By identifying which features are shared versus unique among sample types, subsequent investigations can be focused on a refined subset of annotated features. From this point, statistical analyses and further visualization can be performed, such as evaluating pixel intensities of annotated features in proprietary software tools such as SCiLS, MSI Reader [30], and open‐source packages such as MALDIquant [31], Cardinal [32], and RmsiGUI [33].

**TABLE 1:** Summary of libraries needed to use the script.

dplyr, tidyr, readxl, openxlsx, tibble	Data manipulation, organization and import and export (xlsx)
ggvenn, ComplexUpset	To create Venn diagrams and UpSet plots for visualizing overlaps and unique features
httr	For HTTP requests
RefMet	To harmonize and classify molecule names into a standardized format
ggplot2	For creating detailed visualizations
RColorBrewer	To generate color palettes for improved graphical representation

## 4 Examples of the Protocol Implementation

In Example S1, we compare sorghum stem sections from two developmental stages, 5‐ and 7‐week‐old (Figure 5A). Annotation results with an FDR of 10% were processed using the described script. A Venn diagram (Figure 5B) revealed 51 common features, 79 unique in the 5‐week sample, and 19 unique features in the 7‐week sample. The full list of unique and shared features, including superclass, class, and subclass information [27] can be found in File S1.

Molecule names from the annotated list were harmonized to a standardized chemical nomenclature, and the resulting list of common names was used for chemical classification (Figure 5C). In untargeted metabolomic experiments, such as MSI, this classification step is valuable because METASPACE annotations rely on molecular formulas, MSM scores, and FDRs [12]. Thus, organizing results into chemical categories provides an opportunity to begin exploring the biological context of the samples. For instance, changes in chemical classes can be determined and proportions of those under specific conditions can be visualized [34].

Figure 5C presents a stacked bar plot comparing the number of annotated molecules classified into chemical superclasses based on the ChemOnt chemical taxonomy [27]. As is common in untargeted metabolomics, a significant proportion of molecules remain unclassified. This is largely because the Metabolomics Workbench does not provide standardized common names for all METASPACE annotations, leaving some classification fields blank in the output data frame. This is illustrated in Figure 5C where the first stacked bar (aqua color) represents unclassified features. Even without complete classification, differences in chemical superclass distribution between the two development stages are apparent. The younger stem sample contains higher number of organic acids and glycerophospholipids consistent with active metabolism, cell division, and cell expansion. By 7 weeks, these metabolites decrease, reflecting a development transition toward tissue maturation and reduced metabolic flux and lower membrane biosynthesis.

For the unclassified molecules, alternative strategies can improve name harmonization and further chemical classification. This step includes using a third‐party chemical translation tool that produces results similar to those in Figure 4 B‐8. Available tools include Classyfire, PubChem [35], the Human Metabolome Database (HMDB) [36], the LIPID MAPS, and the Chemical Translation Service (CTS) [37]. For users seeking deeper chemical classification, we recommend using CTS followed by the Batch Compound Classification (https://cfb.fiehnlab.ucdavis.edu/). Figure S1 demonstrates this workflow.

In Example S2, we compare five kidney biopsy tissue sections representing different health conditions (Figure 6A). Samples were interrogated in positive ion mode to measure lipids, and annotations were generated using the LIPID MAPS, SwissLipids, and HMDB databases. When focusing on lipids, the lipid enrichment tool within METASPACE can be applied; this tool is based on the Lipid Ontology (LION) enrichment analysis platform [38].

In this example, we created an UpSet plot to compare the five samples to visualize the number of unique *m*/*z* features per sample, as well as the shared features among them (intersections).

Analysis of the UpSet plot (Figure 6B) indicates that alongside the 32 common *m*/*z* features, each biopsy sample exhibits a distinct set of unique features. The AKI sample contained 85 unique features, compared with the 63 unique features in the control biopsy. These sample‐specific ions may reflect condition‐associated molecular differences and can serve as starting points for identifying candidate biomarkers.

To prioritize these molecular features, we applied an alternative analysis using the same R‐script. We focused specifically on unique features to each biopsy sample. Figure 6B (pie chart) shows the proportion of unique features by each condition. As previously noted, the AKI sample comprises the highest proportion of unique features (36%), followed by the control samples (27%). Figure S2‐B presents the chemical classification of these unique features per sample, where sphingolipids are highly abundant in both AKI and DKD biopsies, likely because of their role in inflammation and mitochondrial dysfunction, the key points of tubular kidney injury [39].

All features in this example were harmonized and further classified, aided by an additional step of manual curation using online tools (Figure S1). Although time‐consuming, this step may be required when a complete classification is desired. The resulting UpSet plot data are available in the File S2.

It is important to note that the chemical classifications presented here rely on the first molecule name listed in the combined METASPACE output. Therefore, we always recommend carefully reviewing the final dataset to identify potential inconsistencies or incorrect annotations. Although METASPACE provides accurate molecular formulas annotations [22], it does not differentiate between isomers. For this reason, the *moleculeNames* column containing all putative identifications is retained in the final data file.

In Example S3, we present an example of a project submitted to METASPACE to showcase the broad applicability of the script, regardless of the ionization source employed in the experiment. In this published dataset [15], IR‐MALDI was systematically tested in positive ion mode using two solvent conditions, each with and without an ice matrix layer. The standard solvent consisted of 50:50 water:ACN with 0.2% formic acid, whereas the doped solvent included 70 μM NH_4_F. Mouse brain samples were analyzed under all four conditions, and the analyses targeted lipids. Data were uploaded to METASPACE and annotated using LIPID MAPS with a 10% FDR; full experimental details can be consulted in the original publication [15].

Figure 7A shows ion images, for an example feature with a *m*/*z* 369.3516 putatively annotated as 3‐Deoxyvitamin D3 and corroborated by the authors as cholesterol [M + H‐H_2_O]^+^. Figure 7B shows a four‐way Venn diagram illustrating the number of shared and unique features across the four experimental conditions. The doped solvent with an ice matrix produced the highest number of unique features (28.1%), followed by the standard solvent with ice (7.8%). These findings are consistent with the authors' observations as the use of an ice matrix is known to enhance molecular detection [40]. We did not evaluate ion intensities as it is in the original study. However, the total number of features with an ice matrix, supporting the reported enhancement (Figure 7C). The corresponding data are provided in the File S3. As expected, samples analyzed without an ice matrix yielded fewer annotated features, regardless of whether the dopant was included. Overall, this script provides an effective and rapid method for screening and summarizing METASPACE annotations. Additional analyses such as assessing relative abundances or spatial intensity patterns across tissue are not currently implemented and remain outside the scope of this workflow. However, because the code is open source, users are encouraged to adapt and extend it to meet their specific analytical needs.

## 5 Conclusion

This protocol provides a user‐friendly R‐script that is particularly accessible for researchers new to MSI data analysis. The script offers clear advantages for untargeted MSI‐based spatial metabolomic experiments, especially when comparing different sample types or treatment groups. It enables a rapid overview of shared and unique annotations from METASPACE and organizes results for downstream biological interpretation through chemical classification. The R‐script is publicly available, and we encourage customization to expand and enhance its functionalities. Adding additional steps, libraries, and functions will further enhance the depth and flexibility of this initial MSI analysis workflow.

## Conflicts of Interest

The authors declare no conflicts of interest.

## Supporting information




**Table S1:** Samples analyzed and presented in Experiment 1.
**Table S2:**. Samples analyzed and presented in Experiment 2.
**Table S3:**. Samples analyzed and presented in Experiment 3.
**Figure S1:**. Step‐by‐step guide for a chemical translation and classification using the Chemical Translation Service (CTS) and the batch compound classification.
**Figure S2:**. Chemical classification of the unique features in the kidney samples.
**File S1:**. Results_Example1.
**File S2:**. Results_Example2.
**File S3:**. Results_Example3.
**Video S1:** Example of R‐script usage.MP4.

**Data S1:** Supporting information.

**Data S2:** Supporting information.

**Data S3:** Supporting information.

## Data Availability

Detailed information regarding the MSI samples, acquisition method, nomenclature in the Supporting Information. MALDI‐MSI data are available on METASPACE: Example S1 Project: https://metaspace2020.org/project/sorghum. Example S2 Project: NIH KPMP (Kidney Precision Medicine Project). Example S3 Project Hunter et al. (2025) Psychosine Detection [15]. The R‐script is available on GitHub https://github.com/AbigailMoreno88/MSI_R and contains instructions to install libraries and for use of the script. Supporting Information video: RScript_usage‐example.MP4 exemplifies the script usage with Example S1. To install R, RStudio, and RTools use the guide provided at the below link https://bioinformatics.ccr.cancer.gov/docs/rtools/R%20and%20RStudio/2.5_installing_r_on_windows/. On Windows‐based operating systems, installation of RTools is required, as it allows R to build, install, and maintain packages from source code.
