# Supplementary material for: A Step‐by‐Step Protocol From METASPACE to Biological Interpretation
Source: J Mass Spectrom. 2026 Jun 11;61(7):e70072. doi: 10.1002/jms.70072 (PMC13255009; doi:10.1002/jms.70072)
Supplement: Supplementary file 1 — Table S1: Samples analyzed and presented in Experiment 1. Table S2:. Samples analyzed and presented in Experiment 2. Table S3:. Samples analyzed and presented in Experiment 3. Figure S1:. Step‐by‐step guide for a chemical translation and classification using the Chemical Translation Service (CTS) and the batch compound classification. Figure S2:. Chemical classification of the unique features in the kidney samples. File S1:. Results_Example1. File S2:. Results_Example2. File S3:. Results_Example3. Video S1: Example of R‐script usage.MP4. [file JMS-61-e70072-s001.docx]

**Supporting information**

**A Step-by-Step Protocol from METASPACE to Biological Interpretation**

Abigail Moreno-Pedraza^1^, Brittney Gorman^1^, Marija Velickovic^1^, Max Bentelspacher^2^, Jaime Barros^2^, Dusan Velickovic^1^, Christopher R. Anderton^1*^

^1^Earth and Biological Sciences Directorate, Pacific Northwest National Laboratory, Richland, WA, 99354, USA.

^2^Division of Plant Science and Technology, University of Missouri, Columbia, MO, USA.

*Anderton@pnnl.gov

**Content**:

Detailed description of Material and Methods

**Example 1** *Sorghum bicolor* stems

**Example 2** Human kidney biopsies

**Example 3** Mouse brain

Supporting Tables

**Table S1.** Samples analyzed and presented in **Experiment 1**.

**Table S2**. Samples analyzed and presented in **Experiment 2**.

**Table S3**. Samples analyzed and presented in **Experiment 3**.

Supporting Figures

**Figure S1**. Step-by-step guide for a chemical translation and classification using the Chemical Translation Service (CTS) and the batch compound classification.

**Figure S2**. Chemical classification of the unique features in the kidney samples

Supporting Files

**File S1**. Results_Example1

**File S2**. Results_Example2

**File S3**. Results_Example3

**Video** Example of R-script usage.MP4

References

**Detailed description of Material and Methods**

***Example 1****. Sorghum bicolor stems*

Lower stem sections from greenhouse grown *S. bicolor* plants were embedded in 7.5% hydroxypropyl methylcellulose (HPMC) with 2.5% polyvinylpyrrolidone (PVP). The embedded sample were mounted on a cryomicrotome chuck by freezing a small droplet of water and then cut into 20-μm-thick sections using a CryoStar NX70 (Thermo Fisher) with a blade temperature of −16 °C and specimen temperature of −14 °C. Tissue sections were thaw mounted on indium tin oxide (ITO) slides and coated with N-(1-naphthyl) ethylenediamine dihydrochloride (NEDC) matrix. Matrix application was performed using an M5 Sprayer (HTX Technologies, Chapel Hill, NC, USA). NEDC was prepared at a concentration of 7 mg/mL in 70% MeOH and was sprayed at 120 µL/min flow rate. The nozzle temperature was set to 70 °C, with 8 cycles at 3 mm track spacing with a crisscross pattern. No drying period was added between cycles, linear flow was set to 1200 mm/min with 10 PSI of nitrogen gas, and a 40 mm nozzle height.

Samples were analyzed with a 12-Tesla SolariX Fourier transform ion cyclotron resonance mass spectrometer (FTICR-MS; Bruker Daltonics) using a spatial resolution of 50 µm. The method employed was specific to detect small molecules and lipids in negative polarity, *m*/*z* range 92–800, an estimated resolving power of 110,000 at *m*/*z* 400, laser power of 40, and 200 laser shots. Imaging data files generated by the FTICR-MS analysis were processed and converted using SCiLS software (Bruker Daltonics) and subsequently exported into the imzML open format for annotation in METASPACE using a mass-to-charge (m/z) tolerance of 3 ppm with the Original MSM Analysis. For this tutorial example: 5-weeks (a-5w-unlabed_neg) and 7-weeks (b-7w-unlabeled_neg) old samples were analyzed compared.

***Example 2****. Human kidney biopsies*

Tissues were sectioned at 7 µm thickness^1^, thaw-mounted on ITO slides, and coated with the matrix 2,5-dihydroxybenzoic acid (DHB). The matrix application was performed using an M5 Sprayer. DHB was prepared at a concentration of 40 mg/mL in 70% MeOH and was sprayed at 50 µL/min flow rate. The nozzle temperature was set to 70 °C, with 12 cycles at 3 mm track spacing with a crisscross pattern. A 2-second drying period was added between cycles. A linear flow of 1200 mm/min was set with 10 PSI of nitrogen gas and a 40 mm nozzle height.

Samples were analyzed with a 12-Tesla SolariX FTICR-MS (Bruker Daltonics) using a spatial resolution of 25 µm. Method was specific to detect lipids in positive polarity, *m*/*z* range 400–1200, estimated resolving power of 160,000 at *m/z* 400, laser power of 40, and 200 laser shots. Imaging data files generated by the FTICR-MS analysis were processed and converted using SCiLS software (Bruker Daltonics) and exported into the imzML open format for annotation in METASPACE using a mass-to-charge (*m*/*z*) tolerance of 3 ppm with the Original MSM Analysis. For this second example we used the following samples: S-2407-021118 (Acute Kidney Injury, AKI), S-2310-016664 (Diabetic Kidney Disease, DKD), S-2212-003371 (Diabetes mellitus resilient, DM-R) S-2305-004587 (Hypertensive- Chronic Kidney Disease, H-CKD) and QC-077 (Control) were compared.

***Example 3****. Mouse brain tissue*

Published datasets from Hunter *et al.*^2^ were downloaded from METASPACE to showcase the utility of the R-script using a different ionization source. IR- MALDESI was used, comparing four analysis methods, doped solvent and ice matrix and their counterparts without. The database LIPID MAPS (LMSD)^3^ was used for annotations. Specifics about this project can be found in the original publication^2^.

**Supplemental Tables and Figures**

**Table S1.** Samples analyzed and presented in **Example 1**. Cryosections from *Sorghum bicolor* stem*.* METASPACE project *Sorghum Stem L and NL.*

| **Sample ID** | **Sample Type** | **METASPACE LINK** |
| --- | --- | --- |
| a-5w-unlabeled_neg | 5-weeks old | <https://metaspace2020.org/dataset/2025-07-15_23h04m13s> |
| b-7w-unlabeled_neg | 7-weeks old | <https://metaspace2020.org/dataset/2025-07-15_23h04m49s> |

**Table S2.** Samples analyzed in **Example 2**.  *Homo sapiens* biopsies from five different conditions. METASPACE projects: NIH Kidney Precision Medicine Project (KPMP) and NIH KPMP Biopsies <https://www.kpmp.org>.

| **Sample ID** | **Abbreviation** | **Biopsies type** | **METASPACE LINK** |
| --- | --- | --- | --- |
| S-2407-021118 | AKI | Acute Kidney Injury | <https://metaspace2020.org/dataset/2025-04-21_23h42m03s> |
| S-2310-016664 | DKD | Diabetic Kidney Disease | <https://metaspace2020.org/dataset/2025-04-21_21h36m11s> |
| S-2212-003371 | DM-R | Diabetes Mellitus Resilient | <https://metaspace2020.org/dataset/2025-04-21_21h34m48s> |
| S-2305-004587 | H-CKD | Hypertensive Chronic Kidney Disease | <https://metaspace2020.org/dataset/2025-07-25_22h41m22s> |
| QC-077 | Control | Healthy Reference Sample | <https://metaspace2020.org/dataset/2025-03-29_00h34m20s> |

**Table S3.** Datasets analyzed in **Example 3** were obtained from METASPACE under the project Hunter et al. (2025) Psychosine Detection^2^.

| **Sample ID** | **Analysis conditions** | **METASPACE LINK** |
| --- | --- | --- |
| Standard w/o Ice | Standard spray without ice as matrix | <https://metaspace2020.org/dataset/2025-07-30_16h56m53s> |
| Twitcher Replicate 4 | Standard spray with ice as matrix | <https://metaspace2020.org/dataset/2025-07-30_17h08m26s> |
| NH_4_F Doped w/o Ice | Solvent doped with NH4F without ice as matrix | <https://metaspace2020.org/dataset/2025-07-30_14h28m34s> |
| NH_4_F Doped w/Ice | Solvent doped with NH4F with ice as matrix | <https://metaspace2020.org/dataset/2025-07-30_16h43m20s> |

**
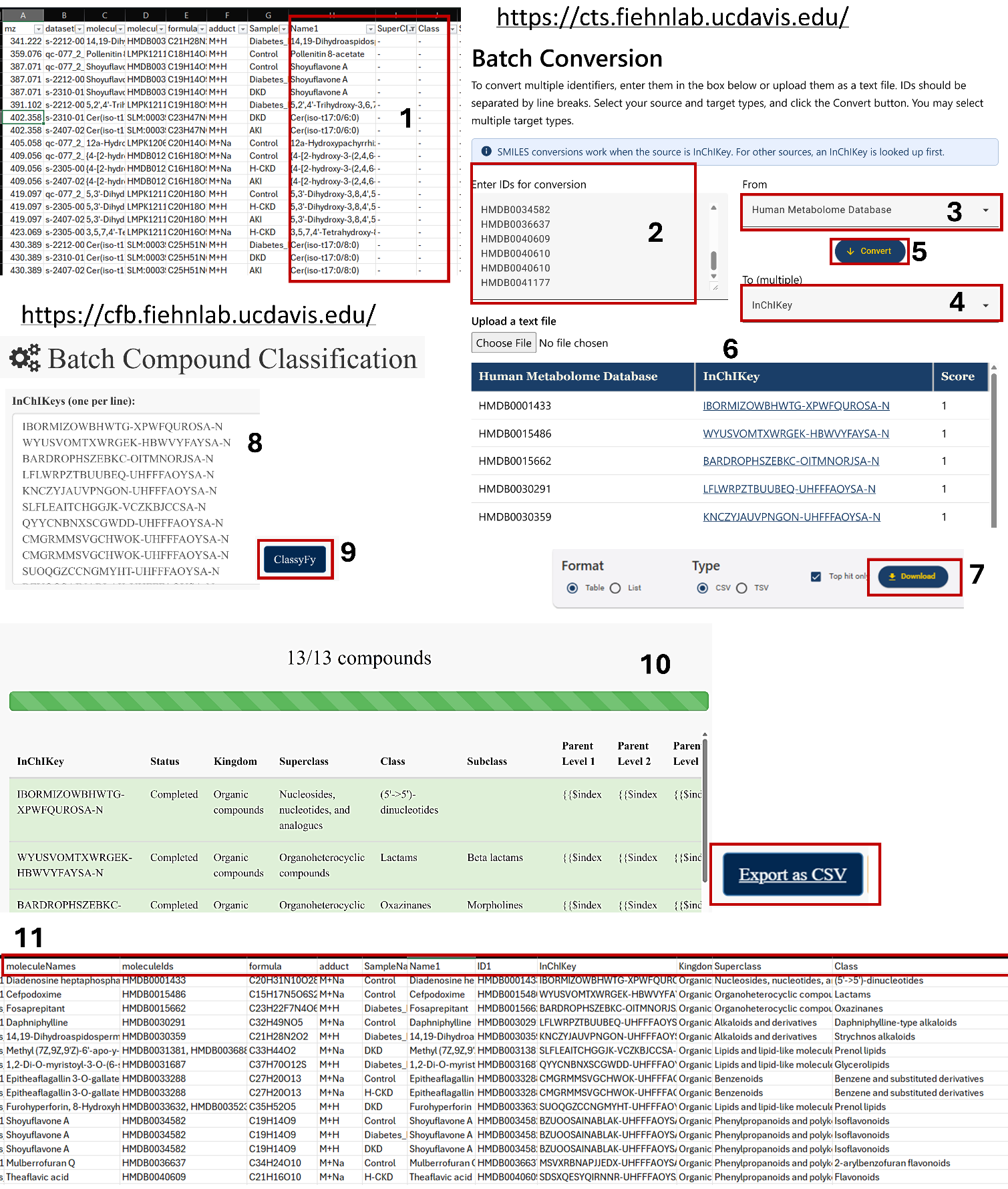
**

**Figure S1**. Step-by-step guide for a chemical classification using the Chemical Translation Service (CTS)^4^ and compound classification using the Batch Compound Classification^5^ FiehnLab batch highlighting the exact steps 1) from the CSV where chemical classes are missing, 2) Select and paste IDs, 3) select the origin of these IDs for example Human Metabolome Database^6^ and 4) the output format desire (InchIKey), 5) click 6) Translation in progress, 7) Download results in a CSV format. Open the Batch Compound Classification website and 8) paste the InchIKey select 9) Classify button, 10) classification in progress, and export results. 11) Compile the new information in the file (ChemicalTranslation.csv). Submit into the R-script for visualization.


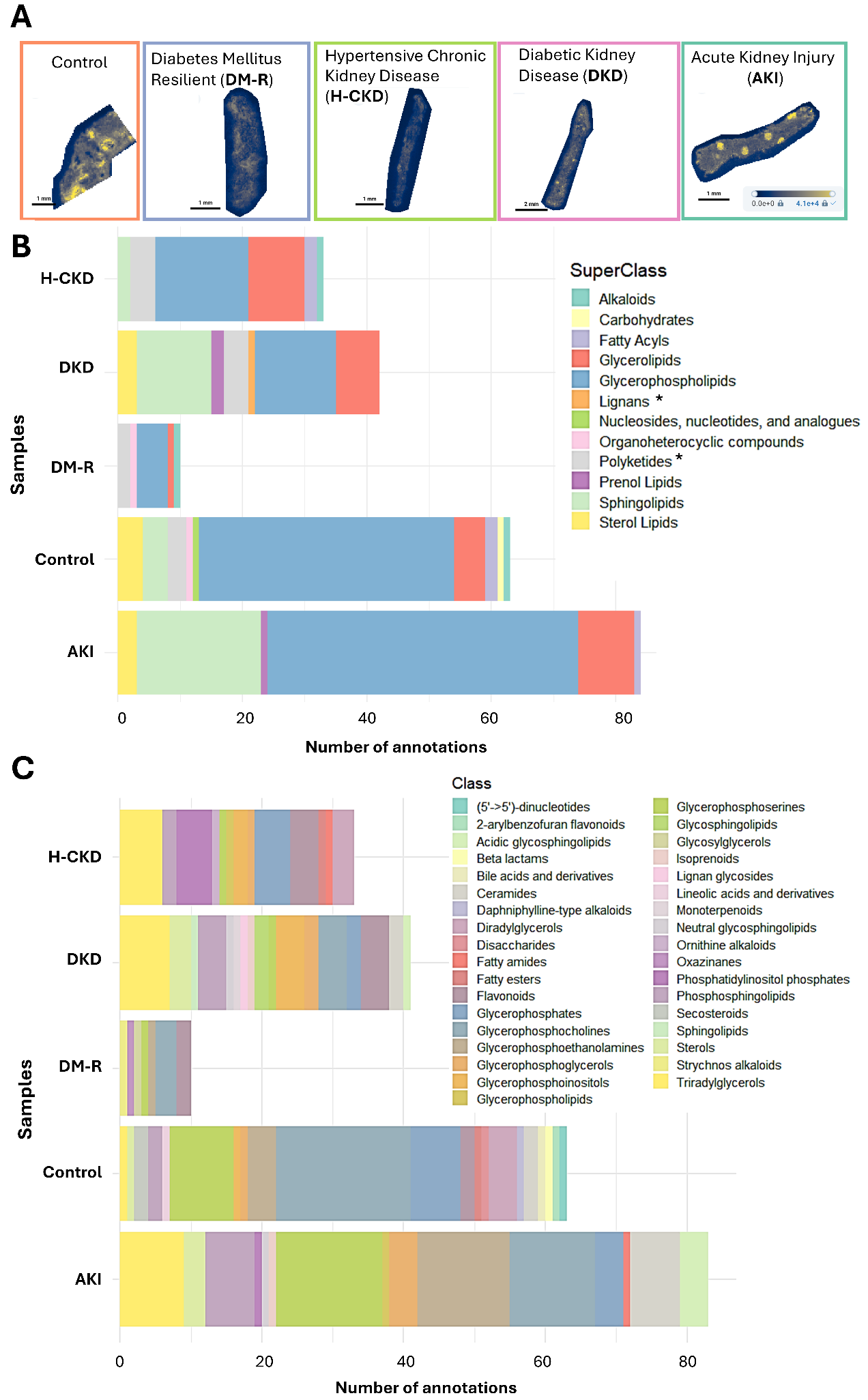


**Figure S2.** Spatial lipidomic analysis across kidney disease groups and chemical-class composition of annotations. **A)** Representative kidney tissue images for each cohort: Control, Diabetes Mellitus Resilient (DM‑R), Hypertensive Chronic Kidney Disease (H‑CKD), Diabetic Kidney Disease (DKD), and Acute Kidney Injury (AKI). **(B)** Stacked bar plots showing the number of molecules annotated and their superclass classification annotations where the superclass sphingolipids may be highly abundant in both AKI and DKD because of their role in inflammation signaling^7^. **C)** Stacked bar plots showing the number of molecules annotated and their chemical classes. An asterisk is placed next to two Superclasses that may be incorrectly annotated in METASPACE or that may be a byproduct of the diet.

**References**

(1) Zhang, G.; Gorman, B.; Hejazi, L.; Velickovic, D.; Alexandrov, T.; R Anderton, C.; Sharma, K. Tissue Preparation for Spatial Metabolomics V.2 In *protocols.io*, 2025.

(2) Hunter, S. N.; Wang, M. F.; Thomas, B. N.; Filiano, A. J.; Muddiman, D. C. Spatially resolved lipids in a mouse brain model of globoid cell leukodystrophy via IR-MALDESI MSI and parallel reaction monitoring MSI. *Anal Bioanal Chem* **2026**, *418* (7), 1973–1986. DOI: 10.1007/s00216-026-06326-3 From NLM Medline.

(3) Sud, M.; Fahy, E.; Cotter, D.; Brown, A.; Dennis, E. A.; Glass, C. K.; Merrill, A. H., Jr.; Murphy, R. C.; Raetz, C. R.; Russell, D. W.; et al. LMSD: LIPID MAPS structure database. *Nucleic Acids Res* **2007**, *35* (Database issue), D527–532. DOI: 10.1093/nar/gkl838 From NLM Medline.

(4) CTS - The Chemical Translation Service. <https://cts.fiehnlab.ucdavis.edu/>.

(5) Djoumbou Feunang, Y.; Eisner, R.; Knox, C.; Chepelev, L.; Hastings, J.; Owen, G.; Fahy, E.; Steinbeck, C.; Subramanian, S.; Bolton, E.; et al. ClassyFire: automated chemical classification with a comprehensive, computable taxonomy. *J Cheminform* **2016**, *8*, 61. DOI: 10.1186/s13321-016-0174-y From NLM PubMed-not-MEDLINE.

(6) Wishart, D. S.; Feunang, Y. D.; Marcu, A.; Guo, A. C.; Liang, K.; Vazquez-Fresno, R.; Sajed, T.; Johnson, D.; Li, C.; Karu, N.; et al. HMDB 4.0: the human metabolome database for 2018. *Nucleic Acids Res* **2018**, *46* (D1), D608–D617. DOI: 10.1093/nar/gkx1089 From NLM Medline.

(7) Wang, Y.; Wu, L.; Bao, X.; Yang, J.; Xu, M.; Chang, Y.; Liu, Z.; Qin, L.; Gao, M.; Lv, C.; et al. Lipid metabolic dysregulation in diabetic kidney disease: mechanisms, cellular impact, and therapeutic strategies. *Journal of Clinical & Translational Endocrinology* **2026**, *44*. DOI: 10.1016/j.jcte.2026.100436.
